# Predicting nicotine metabolism across ancestries using genotypes

**DOI:** 10.1186/s12864-022-08884-z

**Published:** 2022-09-21

**Authors:** James W. Baurley, Andrew W. Bergen, Carolyn M. Ervin, Sung-shim Lani Park, Sharon E. Murphy, Christopher S. McMahan

**Affiliations:** 1 BioRealm LLC, 340 S Lemon Ave, Suite 1931, 91789 Walnut, CA USA; 2 Oregon Research Institute, 3800 Sports Way, 97477 Springfield, OR USA; 3 University of Hawaii, 701 Ilalo Street, 96813 Honolulu, HI USA; 4 University of Minnesota, 2231 6th St SE, 55455 Minneapolis, MN USA; 5 Clemson University, 220 Parkway Drive, 29634 Clemson, SC USA

**Keywords:** Nicotine metabolism, Smoking cessation, Machine learning, Statistical learning, Polygenic risk score, Nicotine biomarkers

## Abstract

**Background:**

There is a need to match characteristics of tobacco users with cessation treatments and risks of tobacco attributable diseases such as lung cancer. The rate in which the body metabolizes nicotine has proven an important predictor of these outcomes. Nicotine metabolism is primarily catalyzed by the enzyme cytochrone P450 (CYP2A6) and CYP2A6 activity can be measured as the ratio of two nicotine metabolites: *trans*-3’-hydroxycotinine to cotinine (NMR). Measurements of these metabolites are only possible in current tobacco users and vary by biofluid source, timing of collection, and protocols; unfortunately, this has limited their use in clinical practice. The NMR depends highly on genetic variation near *CYP2A6* on chromosome 19 as well as ancestry, environmental, and other genetic factors. Thus, we aimed to develop prediction models of nicotine metabolism using genotypes and basic individual characteristics (age, gender, height, and weight).

**Results:**

We identified four multiethnic studies with nicotine metabolites and DNA samples. We constructed a 263 marker panel from filtering genome-wide association scans of the NMR in each study. We then applied seven machine learning techniques to train models of nicotine metabolism on the largest and most ancestrally diverse dataset (N=2239). The models were then validated using the other three studies (total N=1415). Using cross-validation, we found the correlations between the observed and predicted NMR ranged from 0.69 to 0.97 depending on the model. When predictions were averaged in an ensemble model, the correlation was 0.81. The ensemble model generalizes well in the validation studies across ancestries, despite differences in the measurements of NMR between studies, with correlations of: 0.52 for African ancestry, 0.61 for Asian ancestry, and 0.46 for European ancestry. The most influential predictors of NMR identified in more than two models were rs56113850, rs11878604, and 21 other genetic variants near *CYP2A6* as well as age and ancestry.

**Conclusions:**

We have developed an ensemble of seven models for predicting the NMR across ancestries from genotypes and age, gender and BMI. These models were validated using three datasets and associate with nicotine dosages. The knowledge of how an individual metabolizes nicotine could be used to help select the optimal path to reducing or quitting tobacco use, as well as, evaluating risks of tobacco use.

**Supplementary Information:**

The online version contains supplementary material available at 10.1186/s12864-022-08884-z.

## Background

Tobacco smoking is a leading cause of global preventable disease and death. Nicotine, the component of tobacco that sustains nicotine addiction, makes tobacco smoking highly addictive and difficult to quit. Nicotine is primarily metabolized by the cytochrome P450 2A6 (CYP2A6) enzyme. Individual variations in CYP2A6 activity have been found to influence smoking behaviors [1]. A biomarker for measuring CYP2A6 enzymatic activity is the nicotine metabolite ratio (NMR), the ratio of two nicotine metabolites, *trans*-3’-hydroxycotinine (3HC) to cotinine (COT) [2]. The NMR has been shown to be associated with smoking behaviors [1], smoking dose and risk of lung cancer [3], alcohol consumption [4], and smoking cessation [5]. As a result, there have been repeated calls for screening based on the NMR [6].

There remain technical challenges in measuring nicotine metabolism that limits its potential clinical use. Ideally measurement would involve controlled nicotine administration trials which is simply not feasible in large population based studies or in screening. Biochemical measurement of the NMR requires serum, plasma, saliva, or urine for analyte analysis. While measurements of nicotine metabolites have good reproducibility within these biofluids [7], differences in metabolite measurements (e.g. total or free 3HC and COT) make results difficult to compare and interpret across studies [8]. Biochemical measurement of the NMR also requires biofluids to be collected relatively soon after the intake of nicotine, which is impractical in former smokers or if a smoker is an occasional user of tobacco. This has limited NMR-based risk assessments of tobacco attributable diseases and comorbidities to current smokers.

Genomic prediction of the NMR is a promising alternative to direct biochemical measurements. Genotyping services are widespread and feasible within clinical laboratories. A number of functional variants of *CYP2A6* have been shown to be associated with the NMR [9]. More recently genome-wide genotyping have identified additional variants that are associated with the NMR [10–13]. Genomic data has been used to build more comprehensive genomic models [14] and polygenic risk scores [15] for predicting nicotine metabolism. But these models do not generalize across ancestries, requiring the development of ancestry-specific or transferable risk scores [16].

In this work we present the development and validation of an ensemble of models trained to predict NMR using genotypes and basic covariates across ancestries. We begin by prioritizing genetic markers found to be associated with the NMR in four multiethnic studies. We then apply an ensemble of machine learning algorithms to the largest study to train models which are then assessed directly in the other three by comparing observed to predicted NMR. The resulting selected variables and validated models can be used to assess nicotine metabolism in current or former tobacco users. This knowledge can help inform clinical decisions making on the optimal path to smoking cessation and communicate risks for tobacco-related outcomes.

## Methods

### Source of data

Four studies were identified with measured NMR or metabolite data, genomic data or DNA samples available for genotyping, and basic demographic variables. These four studies were used for training and validation of NMR models. They are summarized below. Given the differences in the study designs, including how nicotine metabolites were collected and measured, the focus of this work is on training a predictive model of NMR using the largest and most ancestrally diverse study (the Multiethnic Cohort, MEC). Once the model has been trained, we use the three remaining studies to conduct validation trials by comparing predicted NMR to the measured NMR.

#### Multiethnic Cohort, MEC

The MEC was established in Hawaii and California (primarily Los Angeles County) to study diet and cancer in the United States [17, 18]. From 1993 to 1996, individuals of both sexes, aged 45-75, and from five major racial/ethnic groups (Latino, African-American, Japanese-American, White, Native Hawaiian) were recruited. Participants completed a baseline questionnaire of demographic characteristics, anthropometrics, smoking history and other lifestyle factors. This study uses a subcohort of 2,239 lung cancer free participants who were current smokers at time of biospecimen collection [19]. Collected biospecimens include blood and urine (overnight for Hawaii or first morning in California). Nicotine metabolites were quantified using liquid chromatography-tandem mass spectrometry [20]. Total nicotine equivalents (TNE) was defined as the sum of total nicotine, total COT, total 3HC, and nicotine N-oxide. Here total refers to the sum of the compound and its glucuronide conjugate. The NMR was defined as the urinary total 3HC to free COT ratio.

#### Center for the Evaluation of Nicotine in Cigarettes, CENIC

CENIC conducted studies of the effects of reduced nicotine cigarettes on smoking outcomes. 550 participants across eight United States institutions were randomized to one of seven nicotine levels between June 2013 and July 2014 [21] and had DNA available for analysis. Participants were adult daily smokers that smoke an average of at least five cigarettes per day for at least one year and had either a cotinine (COT) level $> 100$ ng/mL or expired carbon monoxide (CO) of $>8$ ppm [21]. Smokers were initially assessed using their usual brand cigarettes. Nicotine metabolites levels, including COT, were measured using liquid chromatography with tandem mass spectrometry and expired CO levels were measured using a Smokerlyzer ED50 by Bedfont Instruments [21]. TNE was defined as the sum of total nicotine, total COT, total 3HC, and nicotine N-oxide. The NMR was defined as the urinary total 3HC to free COT ratio.

#### Hawaii Smokers Study, HSS

The HSS was comprised of 600 participants randomly selected from the MEC participants who were current smokers at time of study, reporting that they smoked at least 10 cigarettes per day, had no history of cancer, and were self-reported Japanese, European, or Hawaiian ancestry [22]. Study interview and blood and 12-hour urine samples were collected independent of the previously mentioned MEC biospecimen collection. Analysis of total urinary nicotine, COT, and 3HC concentration was done by GC/MS (gas chromatography/mass spectrometry). Nicotine equivalents (NE) was defined as the sum of total nicotine, total COT, and total 3HC. The NMR was defined as the urinary total 3HC to total COT ratio.

#### Laboratory studies of nicotine metabolism, METS

The METS included 315 unrelated African-American, Asian-American, and European-American individuals from three laboratory studies of nicotine metabolism [12]. The studies included Pharmacokinetics in Twins (PKTWIN) [23], Pharmacogenetic Study of Nicotine Metabolism (588) [24], and SMOking in FAMilies (SMOFAM) [25]. Blood or saliva was collected 6 hours after the administration of labeled nicotine and cotinine in smokers and non-smokers. Nicotine metabolite levels were assessed using gas chromatography-tandem mass spectrometry methods. The NMR was defined as the 6 hour plasma or saliva 3HC to COT ratio.

### Response variable

We aim to develop a predictive model of the urinary nicotine metabolite ratio (the ratio of total 3HC to free COT) from genotypes and covariates.

### Predictors

#### Genotypes

Imputed genotypes were already available for the MEC and METS studies [12, 13]. The MEC was previously genotyped on the Illumina Human1M-Duo BeadChip. The METS were previously genotyped on the Smokescreen Genotyping Array. Both were imputed to include variants in the 1000 Genomes Project reference populations using standard phasing and imputation best practices at the time [12, 13].

DNA samples from HSS and CENIC were genotyped specifically for this project on the Smokescreen Genotyping Array [26]. 200 ng of genomic DNA were plated using Axiom 2.0 Reagent Kits and processed on the GeneTitan MC instrument. Analysis of the raw data was performed using Affymetrix Power tools (APT) v-1.16. Additional steps were performed using SNPolisher to identify and select probe sets and high quality variants for downstream analysis. Quality control steps for samples included comparisons of self-reported and genomic gender and ancestry, detection of excessive heterozygosity ($> 0.20$), genotype concordance among known duplicates, and removal of unexpected duplicates and related samples. Quality control steps for genetic variants included missingness $> 5\%$ and deviation from Hardy Weinberg equilibrium ($p <$1E-10). After quality control, HSS had genotypes for 585 individuals on 569,986 genetic variants. CENIC had genotypes for 515 individuals on 570,258 genetic variants.

We used genome-wide imputation to harmonize genotypes across the studies prior to analysis. Alleles were reported on the forward strand and conform-gt was used to ensure consistency with the 1000 Genomes Phase 2 version 5a data files prepared for use with the Beagle imputation software. Beagle 5.2 was used to phase genotypes and impute ungenotyped or missing genotypes [27]. The resulting genotype dosages for variants typed on the Smokescreen Genotyping Array were imported into a Postgres database.

#### Covariates

We compiled age, sex, self-identified ethnicity, body mass index (BMI), and smoking status (from METS) from study datasets. Additionally, ancestry proportions were estimated by extracting genotypes for 5516 ancestry informative markers from the study data and combining it with genotypes from 1000 Genomes Project Phase 3 version 5a. fastSTRUCTURE was used with default settings and $k=3$ populations [28]. Populations assignments from the 1000 Genome Project and self-reported race from the studies were used to label the estimated European, Asian, and African ancestry proportions.

### Sample size

The NMR was merged with genotypes and covariates for each study to create the analytic dataset. Sample sizes were 2,239 for MEC, 515 for CENIC, 585 for HSS, and 315 for METS.

### Missing data

HSS was missing 5 observations for NMRs and those records were dropped from the analysis. The genome-wide assocation scans of NMR used for marker nomination used complete observations. In model training and validation, missing values were imputed using the missMDA package in R [29]. Briefly, the data for candidate predictors were stacked across studies, the number of dimensions were estimated by principal components analysis (PCA), and the missing values were imputed with the PCA model.

### Statistical analysis methods

#### Marker nomination

Prior to training NMR prediction models, we nominated markers to consider using results from genome-wide association scans. The scans used models of the form

1 $$\begin{aligned} Y_{i}= \mathbf {X}_{i}' \varvec{\beta } + S_{ij}\alpha _{0} + \mathbf {P}_{i} \varvec{\alpha } +\epsilon _{i}, \end{aligned}$$

where $Y_i$ is the natural log NMR measured on the *i*th individual, $\mathbf {X}_i$ is a vector of covariates with $\varvec{\beta }$ being the corresponding vector of regression coefficients, $S_{ij}$ is the genetic variant under consideration with $\alpha _0$ being the corresponding regression coefficient, $\mathbf {P}_i$ is a vector of principal components computed on the genotypes design matrix with $\varvec{\alpha }$ being the corresponding vector of regression coefficients, and $\epsilon _i$ is the error term. In this analysis, we controlled for age, sex, ancestry, body mass index (BMI), and smoking status in the METS. We used the first 50 principal components of the genotype design matrix to account for genetic relatedness/ancestry among the study participants. The *p*-values for the test of $H_0:\alpha _0=0$ vs. $H_1:\alpha _0\ne 0$ were computed.

From these results, we selected 200 genetic variants from each study based on the smallest *p*-values with allele frequencies $> 1\%$. We took the union of these sets and retained genetic variants with evidence ($p < 0.05$) of association with the NMR in at least two of the four studies.

#### Model training and validation

To develop a predictive model of NMR, we took an ensemble based approach that leveraged a suite of machine learning algorithms. This suite consisted of partial least squares [30], projection pursuit [31], elastic net [32], support vector machine (with a linear and radial basis function kernel) [33], gradient boosting machine [34], and random forests [35]. Each of these machine learning models was fit to the MEC data (the largest and most diverse of the four studies), treating the $Y_i$ (NMR measured on the *i*th subject) as the response variable. To explain the heterogeneity in the NMR in this analysis, we used a feature set consisting of age, gender, BMI, Asian and African ancestry proportions, and the 263 prioritized markers arising from the marker nomination step. For notational brevity, we denote the feature set for the *i*th observation by $\mathbf {F}_i$. The R package caret was used to fit and train all of the models using the methods listed in Table 1.

**Table 1:** Fitting method and tuning parameter configurations. Provided are the considered training parameters for partial least squares (PLS), project pursuit (PPR), elastic net (ENet), support vector machine with a linear kernel (SVM-L), support vector machine with a radial basis function kernel (SVM-R), gradient boosting machine (GBM), and random forests (RF). Also provided are the model fitting methods

Model	Tuning Grid	Method
PLS	Number of components $\in \{1,...,20\}$	pls
PPR	Number of terms $\in \{1,..,5\}$	ppr
Enet	Mixing percentage $\alpha \in \{0.10,0.55,1.00\}$	glmnet
	Penalty parameter $\lambda \in \{8.975e^{-4},8.975e{^-3},8.975e^{-2}\}$	
SVM-L	Cost parameter $\in \{0.001,0.002,...,0.02\}$	svmLinear
SVM-R	Cost parameter $\in \{5,...,20\}$	svmRadialSigma
	RBF kernel parameter $\sigma \in \{0.0001,0.0005,...,0.02\}$	
GBM	Interaction depth $\in \{1,...,5\}$	gbm
	Number of trees $\in \{10,20,...,100\}$	
	Shrinkage 0.1	
	Minimum number of Obs. in a node 10	
RF	Number of randomly selected predictors $\in \{1,...,10\}$	rf

**Fig. 1:**
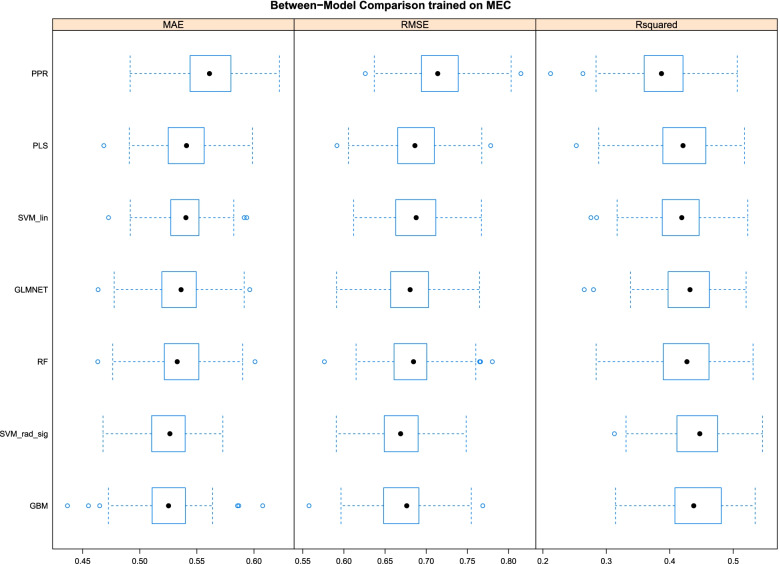
Assessment of the seven models in the training data (MEC). Models were trained using project pursuit (PPR), partial least squares (PLS), support vector machine with a linear kernel (SVM_lin), elastic net (GLMNET), random forests (RF), support vector machine with a radial basis function kernel (SVM_rad_sig), and gradient boosting machine (GBM). Model performances were assessed using mean absolute error (MAE), root mean squared error (RMSE), and R Squared. The boxplots summarizes these metrics across 100 cross validation datasets. Performances were similar across the models justifying use of an average of predictions in the ensemble model

It is important to note that the fitting process for each of the aforementioned models required the selection of tuning parameters that have to be specified in a methodical way to avoid issues of over- and under-fitting the data. Classically, this issue can also be described via the bias-variance tradeoff. That is, a model that is not appropriately regularized, or is overspecified, can over-fit the data thus reducing the bias at the expense of increased variability. In contrast, an underspecified model, or over regularized, could provide for less variable predictions but at the expense of increased bias. To choose the tuning parameters, we implemented repeated (10 times) 10-fold cross validation at an array of candidate tuning parameter values; for more on repeated cross validation see [36]. The grid of tuning parameters were designed based on initial analyses based on default settings, prior experience, and to ensure that the optimal configuration existed on the interior of the grid; i.e, the optimal value did not exist on the boundary of the grid. The optimal tuning parameter configuration for each of the machine learning models was determined to be the one that minimized the cross-validation error. Table 1 summarizes the candidate tuning parameters for each of the machine learning models. Note, our cross validation strategy requires fitting 100 models for each tuning parameter configuration for each of the considered machine learning model. Figure 1 provides a summary of these fits at their optimal tuning parameter configuration. This summary includes the mean absolute error (MAE), the root mean squared error (RMSE), and the R-squared value for each of the 100 fits.

**Fig. 2:**
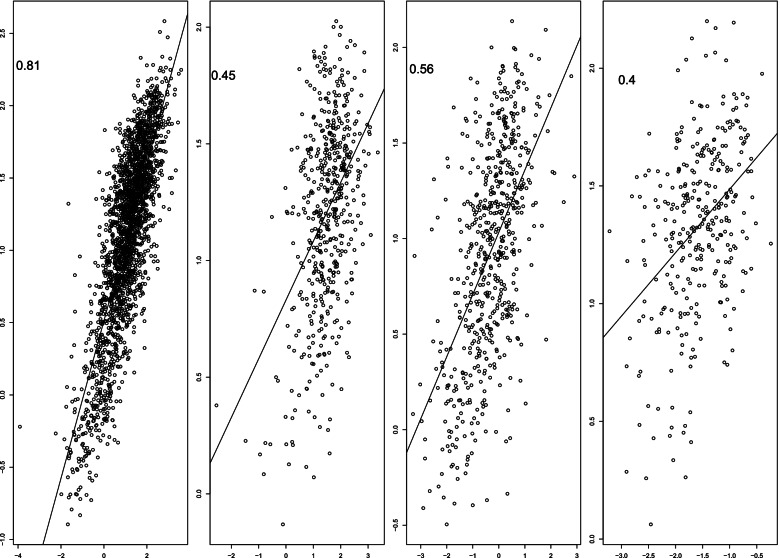
Observed versus predicted NMR values for the training (MEC) and validation (CENIC, HSS, and METS) data. The predicted NMR is the averages of the predictions from the seven models (i.e., the ensemble model). The correlation between these values are displayed to the upper left of each scatterplot. The distribution of NMRs were different across studies, yet the correlations were still strong in the validation datasets

Once the process of training the models was complete, the ensemble model was constructed; for further discussion on ensemble based techniques see [37]. Let $\widehat{f}_j(\cdot )$ denote the *j*th fitted sub-model. Based on these fitted sub-models, our ensemble is given by $\widehat{f}(\cdot ) = 1/7\sum _{j=1}^7\widehat{f}_j(\cdot )$. Thus, the trained model can provide predictions of the NMR ($\widehat{Y}$) for a new feature set ($\mathbf {F}$) as $\widehat{Y}=\widehat{f}(\mathbf {F})$. That is, this ensemble provides predictions by averaging the predictions of the individual sub-models. Proceeding in this fashion we obtain more reliable predictive performance than could be obtained from any one of the component models alone. To examine performance of our trained ensemble, we use it to predict the NMR for the subjects in the CENIC, HSS, and METS studies. Supplementary Fig. S3 provides the predicted vs. the actual NMR across all four studies for each of the seven models and Table 2 provides the correlations between predicted and actual NMR by study and model stratified by estimated genomic ancestry. Figure 2 aggregates the predictions from each model to form an ensemble based prediction.

**Table 2:** Correlations between predicted and observed NMRs by study, ancestry, and model. The correlations between predicted and observed NMRs were summarized overall and by genomic ancestry (ancestry proportion $>0.5$). The MEC was the largest and most diverse sample and was used for training using partial least squares (PLS), project pursuit (PPR), elastic net (ENet), support vector machine with a linear kernel (SVM-L), support vector machine with a radial basis function kernel (SVM-R), gradient boosting machine (GBM), and random forests (RF). Predictions from these seven models were averaged in an ensemble model. The MEC trained models were applied to CENIC, HSS, and METs for validation

	PLS	PPR	ENet	SVM-L	SVM-R	GBM	RF	Ensemble	N
**MEC (Training)**									
African	0.60	0.67	0.58	0.75	0.58	0.76	0.97	0.76	342
Asian	0.71	0.76	0.70	0.79	0.69	0.81	0.97	0.82	995
European	0.50	0.56	0.49	0.63	0.48	0.64	0.97	0.67	902
Overall	0.71	0.75	0.70	0.78	0.69	0.79	0.97	0.81	2239
**CENIC (Validation)**									
African	0.35	0.33	0.32	0.33	0.32	0.41	0.41	0.37	111
Asian	0.71	0.28	0.68	0.60	0.67	0.51	0.50	0.61	9
European	0.42	0.38	0.43	0.41	0.37	0.52	0.47	0.46	395
Overall	0.42	0.37	0.41	0.39	0.37	0.50	0.45	0.45	515
**HSS (Validation)**									
African									1
Asian	0.51	0.29	0.55	0.59	0.53	0.55	0.58	0.56	308
European	0.42	0.33	0.42	0.37	0.36	0.42	0.39	0.43	271
Overall	0.53	0.37	0.56	0.56	0.53	0.55	0.56	0.56	580
**METS (Validation)**									
African	0.43	0.30	0.50	0.55	0.41	0.46	0.45	0.52	48
Asian	0.37	0.03	0.39	0.47	0.41	0.47	0.47	0.43	51
European	0.38	0.10	0.40	0.45	0.42	0.44	0.41	0.43	216
Overall	0.36	0.09	0.42	0.42	0.39	0.45	0.45	0.40	315

## Results

### Participant characteristics

The characteristics of the participants for each study are presented in Table 3. The MEC and HSS individuals were older on average than CENIC and the METS (mean ages of 64, 61, 43, and 34 respectively). Participants of CENIC had a greater proportion of males than the other studies (59% versus 49% (HSS), 45% (METS), and 46% (MEC)) and larger body mass. The METS study had a mix of smokers (45%) and nonsmokers, whereas the other study participants were all smokers.

**Table 3:** Participant characteristics for the training data (MEC) and the three validation datasets (CENIC, HSS, and METS)

	MEC	CENIC	HSS	METS
	(N=2239)	(N=515)	(N=580)	(N=315)
**Age**				
Mean (SD)	63.9 (7.19)	43.4 (13.2)	60.6 (9.40)	33.8 (10.9)
Median [Min, Max]	63.0 [45.0, 86.0]	44.0 [18.0, 75.0]	60.6 [19.6, 83.2]	30.0 [18.0, 69.0]
**Body Mass Index**				
Mean (SD)	26.3 (5.31)	30.0 (6.70)	27.1 (6.10)	25.6 (4.75)
Median [Min, Max]	25.6 [11.3, 62.8]	29.2 [15.2, 56.0]	26.1 [14.4, 54.8]	24.9 [15.9, 49.1]
Missing	0 (0%)	3.00 (0.5%)	0 (0%)	0 (0%)
**Gender**				
Male	1040 (46.4%)	306 (59.4%)	284 (49.0%)	141 (44.8%)
Female	1199 (53.6%)	209 (40.6%)	296 (51.0%)	174 (55.2%)
**Current Smoker**				
Yes	2239 (100%)	515 (100%)	580 (100%)	120 (38.1%)
No	0 (0%)	0 (0%)	0 (0%)	195 (61.9%)
**Self Reported Race**				
African American	364 (16.3%)	107 (20.8%)	0 (0%)	49 (15.6%)
American Indian/Alaskan Native	0 (0%)	5 (1.0%)	0 (0%)	0 (0%)
Asian American	0 (0%)	6 (1.2%)	0 (0%)	51 (16.2%)
Multirace	0 (0%)	25 (4.9%)	0 (0%)	0 (0%)
White	437 (19.5%)	372 (72.2%)	197 (34.0%)	215 (68.3%)
Japanese American	674 (30.1%)	0 (0%)	191 (32.9%)	0 (0%)
Native Hawaiian/Pacific Islander	311 (13.9%)	0 (0%)	192 (33.1%)	0 (0%)
Latino	453 (20.2%)	0 (0%)	0 (0%)	0 (0%)
**Natural log Nicotine Metabolite Ratio**				
Mean (SD)	1.11 (0.898)	1.50 (0.720)	-0.324 (0.959)	-1.58 (0.548)
Median [Min, Max]	1.20 [-3.91, 3.60]	1.54 [-2.54, 3.35]	-0.267 [-3.30, 2.90]	-1.57 [-3.22, -0.240]

The distribution of self-reported races varied by study and as expected corresponded to estimated ancestry proportions (see Supplementary Table S1). The MEC included African American (16%), Japanese American (30%), Native Hawaiian/Pacific Islander (14%), Latino (20%), and White (20%) smokers. The METS were comprised of self-reported African American (16%), Asian American (16%), and White (68%) participants. The HSS had nearly equal proportions of Japanese American, Native Hawaiian/Pacific Islander, and White smokers. The smokers in CENIC were mostly White (72%) and African American (21%).

The distributions of natural log NMRs varied by study (Supplementary Fig. S1) and represented differences in collection source and timing, metabolite measurements, and study patient characteristics. MEC reported the urinary total 3HC to free COT in smokers; CENIC reported urinary total 3HC to free COT in smokers; HSS reported urinary total 3HC to total COT in smokers; and METS reported plasma or salivary 3HC to COT at 6 hours after a fixed dose of nicotine was administered. This precluded stacking the data for model training.

### Model development

The distribution of marginal *p*-values found in the four genome-wide association scans of NMR are provided in Supplementary Fig. S2. There does not appear to be any inflation or deflation of the *p*-values overall (i.e. $\lambda$’s are close to one). In each study, there were many genetic variants with small *p*-values; as seen in the the tail of the distributions presented in Supplementary Fig. S2. The smallest 200 *p*-values with allele frequencies $> 1\%$ were merged across the studies. After filtering (see Section 2.6.1), there were 263 genetic variants selected as candidates predictors of NMR. The list of markers and their corresponding chromosome, position, and alleles can be found in the Supplementary Files. MEC had the largest and most diverse sample of the studies considered. Given this, we trained the models on the MEC dataset and validated them in the other three studies.

### Model performances

Within MEC, model performances were summarized across 100 model fits at their optimal tuning parameter configuration which are presented in Fig. 1. These included the mean absolute error (MAE), root mean squared error (RMSE), and the R-squared. Models with lower values of MAE and RMSE achieve higher model accuracy. The R-squared is the proportion of variance explained by the model. As shown in Fig. 1, the trained models can explain about half of the variability in NMR. The performances across the models are remarkably similar, with no clear winner or loser. Given this observation, we give each model equal weights in the ensemble model. That is, the ensemble model is simply the average of the predicted NMR values from the seven component models.

The model performances within sample (MEC) and out-of-sample (HSS, METS, CENIC) for each model and the overall ensemble are presented in Supplementary Fig. S3 and Fig. 2 respectively. Here out-of-sample refers to data that the model was not trained on, and represents a setting that offers an unbiased assessment of overall performance. Within MEC, the correlations between the observed and predicted NMR ranges from 0.69 to 0.97 depending on the model. The correlation of the ensemble and observed values is 0.81 indicating that averaging the predictions from the member models yields good prediction performance. The models also generalizes well out-of-sample. The correlation between the observed NMR and the predicted NMR from the ensemble are 0.45, 0.56, and 0.4 for CENIC, HSS, and METS respectively (Fig. 2).

The correlations for each component model stratified by ancestry are presented in Table 2. Overall, the ensemble model performed well across ancestries in the MEC (0.76, 0.82, and 0.67 for African, Asian, and European ancestries respectively). In the validation studies, the best ensemble correlations for African ancestry was 0.52 in the METS, for Asian ancestry was 0.61 in CENIC, and for European ancestry was 0.46 in CENIC.

### Variable importance

**Fig. 3:**
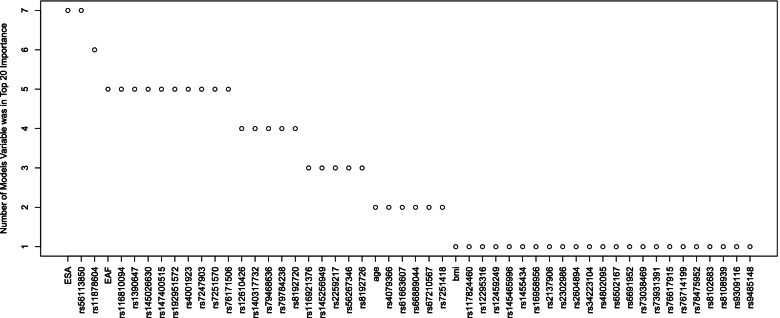
Summary of the most important candidate variables in the NMR models. Variable importance was ranked for each of the seven models trained on the MEC data. The number of times each variable occurred in the top 20 for each model was enumerated. Asian ancestry and rs56113850 was an influential variable in all the models

Variable importance is an indicator of how much a candidate predictor contributes to a model on a scale of zero to 100. These are presented for the seven models trained in the MEC data in Supplementary Fig. S4. In examining the patterns, there was consensus among the models on the importance of many of the variables in predicting NMR yet diversity in the variables each models deemed important. Given this observation and the performances of the models being rather similar, the panel of 263 genetic variants seems adequate. We next examine the favorites by ranking variables by importance for each model and counting how many times each variable occurs in the top 20 (Fig. 3). Asian ancestry and rs56113850 were highly relevant to the prediction of NMR in all models. rs11878604 was also important in predicting NMR (six models). Additionally, African ancestry, age, and 24 genetic variants were of top importance in more than two models.

## Discussion

The NMR is an important biomarker for selecting the optimal intervention for smokers seeking to quit and for evaluating the risks of tobacco use. We selected a panel of 263 genetic markers from genome-wide analyses in four multiethnic dataset, trained the models in the largest, most ancestrally diverse dataset, and validated models in three additional multiethnic datasets. In summary, we have created an ensemble model for estimating the NMR across ancestries from genotypes, genetic ancestry, and basic individual characteristics (i.e., age, height, weight, and gender). This work also provides a methodological framework for developing other genomic-based assessments of heritable biomarkers.

Diversity was a strengths in our approach and findings. The American individuals used in model training and validation studies represented diverse ancestries, composed of Asian, African, and European backgrounds. This enabled us to detect genetic variants related to nicotine metabolism that we would not have selected otherwise and train models that generalize to new observations. This is highlighted in Table 2 by the out-of-sample performances of the models in CENIC, HSS, and METS across ancestries. We also adopted an ensemble based approach that averages NMR predictions from seven different component models. While performances of these seven models were similar, there was variation in how the candidate variables contribute to the model (Supplementary Fig. S4). This diversity in solutions is a strength, enabling more reliable predictions in the ensemble model.

The most important predictors in these models combine many of the findings in the literature on the genetics of the NMR. Of the 26 genetic variants flagged in more than one model (Fig. 3), 7 had entries in the NHGRI-EBI Catalog of human genome-wide association studies [38]. The marker rs56113850 was associated with nicotine metabolism at genome-wide significance in smokers of European ancestry [10, 11]. In smokers of African ancestry, the marker rs11878604 was associated with NMR at genome-wide significance and identified as an independent signal [39]. This marker was also top ranked in African American female smokers [40]. The marker rs12459249 was associated at genome-wide significance and top ranked in the METS meta-GWAS [26], and was an independent signal identified in conditional analysis of African American treatment seeking smokers [39]. Markers rs11878604 and rs116921376 have been implicated in tobacco-related consequences (lung cancer and chronic obstructive pulmonary disease) [41]. The marker rs56267346 has been identified as playing a role in caffeine metabolism, which involves *CYP2A6* [42]. rs8192726 has been shown to be related to cigarettes per day in a genomic study of 1.2 million individuals [43]. These variants are all located at or near the *CYP2A6* gene on chromosome 19. Age and BMI have previously been shown to influence the NMR in treatment-seeking smokers [44]. Genomic estimated Asian and African ancestries were important in five and seven models, respectively, and have been shown to influence nicotine metabolism [45].

### Limitations

Differences in NMR measurements prevented us from stacking the data across studies. These differences included patient characteristics (type of smoker, nicotine administration, age, ancestry, etc.), biospecimens collected (urine, blood, saliva), the timing of collection (steady state kinetics or laboratory sampling), and the metabolite measurement procedures. For example, in the METS, participants were administered fixed doses of labeled nicotine and cotinine followed by biospecimen collection at regular intervals, with the 6 hour collection being used to assess NMR. However, this timing does not allow the metabolite ratio to reach a steady state, and thus the NMR is under estimated relative to NMR measured based on steady state kinetics as in the MEC [8]. In addition, non-smokers will have slightly reduced NMR estimates compared to smokers, as cigarette smoking has been associated with increases in the NMR [44]. Thus, the NMR of the non-smokers in METS is an underestimate of the steady-state NMR in smokers of the same genomic background. This may account for the lower correlation between the predicted urinary total 3HC/free COT and the laboratory-based NMR in the METS. Additionally, the HSS NMR measure included total cotinine, with individual variation in the cotinine glucuronidation ratio, as this NMR measure contains glucuronidated cotinine in the denominator [8, 46]. Specific adjustment for cotinine glucuronidation ratio was shown to substantially improve prediction of plasma NMR using the urinary total 3HC to total COT ratio [3, 8]. These differences however provided a unique opportunity for validation since while the NMR measures do differ between some of the cohorts studied here, we do know these NMRs are correlated [8, 46]. As such, in our analysis, we trained on the largest and most diverse cohort (MEC), and were able to validate the NMR models in the other three studies (METS, CENIC, and HSS). While the correlation between the observed and predicted NMRs were the strongest in the training set as expected (0.81 for the MEC ensemble model), the predicted NMRs also correlated with measured NMRs in the validation sets (0.45, 0.58, and 0.40 for CENIC, HSS, and METS respectively).

As noted, we trained the models on the largest multiethnic sample. However, MEC was genotyped using an older genotype array (Illumina Human1M-Duo BeadChip), while the other studies were genotyped with the Smokescreen Genotyping Array, designed with more markers within gene regions related to nicotine metabolism and smoking-related behaviors outcomes [14, 26]. While all studies were imputed to the 1000 Genomes Project, there were more poor quality genotypes (typed or imputations) in the older studies MEC (23 markers) and METS (28 markers) than the newly genotyped studies (5 markers in CENIC and 12 markers in HSS).

### Validation with smoking dosage in the HSS and CENIC

The rate of nicotine metabolism influences how much nicotine an individual is exposed to (i.e., nicotine dosage) and consequently risk of lung cancer [3]. Nicotine equivalents is the sum of nicotine and nicotine metabolites, and offers a more precise measure of nicotine intake than self-reported cigarettes per day. To link predicted nicotine metabolism to nicotine exposure, we took the NMR predictions for HSS and CENIC using the MEC trained ensemble model, and examined their relationship to nicotine equivalents. We found that the predicted NMRs were strongly associated with nicotine equivalents in both studies ($p=3.3E-4$ and $p=1.6E-7$ in CENIC and HSS respectively). This indicates that predicting how an individual metabolizes nicotine could be used to quantify their nicotine exposure and tobacco-attributable disease risk.

### Implications and future work

Direct measurement of the NMR has its challenges. For example, individuals must be actively using nicotine-containing products and there are issues related to measurement and sample collection. We offer an approach where genotypes and basic demographics could be used to characterize how a current or previous tobacco user metabolizes nicotine. Genotypes could be obtained by inexpensive genotyping platforms and paired with popular saliva DNA collection kits. The knowledge of how an individual metabolizes nicotine could be used to help select the optimal path to reducing or quitting tobacco use, as well as, evaluating risks of tobacco-related diseases and comorbidities.

Additional work is needed to optimize the predictive models using larger population representative samples with genotypes and both plasma and urine nicotine metabolites. Training models on different versions of NMR may improve prediction performance; e.g., NMR measured relative to different metabolite combinations. In optimizing these models, we plan to consider structural variations (e.g., copy number variants, gene duplication, deletions, and translocations) for genes involved in the nicotine metabolism pathway (e.g., *CYP2A6* and *UGT2B10*). These future models should also consider environmental factors that influence NMR, e.g., estrogen, comorbidites and diet [1, 44, 47] as well as additional components of the nicotine metabolism pathway (e.g., N-oxidation pathways [48–50]).

However, the presented models may be immediately used to predict nicotine metabolism in newly collected or existing DNA samples, or from existing genomic data. These predictions in turn can be linked to clinical outcomes. For example probabilistic models could be built that relate the predicted NMR to the likelihood of smoking cessation or response to different treatment options. This could lead to the identification of NMR cut-points that could be used to guide subject specific treatment paths. Additionally, our prediction model could help improve the understanding of nicotine metabolism in large representative populations (e.g., Population Assessment of Tobacco and Health [51]). This could help inform proposed and actual regulatory thresholds for nicotine levels.

## Supplementary Information

**Additional file 1.** Supplementary Figures.

**Additional file 2.** Supplementary Table.

**Additional file 3.** List of genetic variants nominated for prediction of nicotine metabolism. This file contains the rs number, chromosome, hg19 position, reference allele, and alternative allele for the 263genetic variants selected as candidates for prediction of the nicotine metabolite ratio.

## Data Availability

The data that support the findings of this study are available from the principal investigators of the individual studies. Restrictions apply to the availability of these data, which were used under license for this study. Data are available from the corresponding author with the permission of the principal investigators of the individual studies.

## References

[1] Benowitz NL, Hukkanen J, Jacob P (2009). Nicotine chemistry, metabolism, kinetics and biomarkers. Handb Exp Pharmacol..

[2] Hukkanen J, Jacob P, Benowitz NL (2005). Metabolism and disposition kinetics of nicotine. Pharmacol Rev..

[3] Murphy SE (2021). Biochemistry of nicotine metabolism and its relevance to lung cancer. J Biol Chem..

[4] Roberts W, Marotta PL, Verplaetse TL, Peltier MR, Burke C, Ramchandani VA (2020). A prospective study of the association between rate of nicotine metabolism and alcohol use in tobacco users in the United States. Drug Alcohol Depend..

[5] Lerman C, Schnoll RA, Hawk LW, Cinciripini P, George TP, Wileyto EP (2015). Use of the nicotine metabolite ratio as a genetically informed biomarker of response to nicotine patch or varenicline for smoking cessation: a randomised, double-blind placebo-controlled trial. Lancet Respir Med..

[6] Siegel SD, Lerman C, Flitter A, Schnoll RA (2020). The Use of the Nicotine Metabolite Ratio as a Biomarker to Personalize Smoking Cessation Treatment: Current Evidence and Future Directions. Cancer Prev Res..

[7] St Helen G, Novalen M, Heitjan DF, Dempsey D, Jacob P, Aziziyeh A (2012). Reproducibility of the nicotine metabolite ratio in cigarette smokers. Cancer Epidemiol Biomarkers Prev..

[8] Giratallah HK, Chenoweth MJ, Addo N, Ahluwalia JS, Cox LS, Lerman C (2021). Nicotine metabolite ratio: Comparison of the three urinary versions to the plasma version and nicotine clearance in three clinical studies. Drug Alcohol Depend..

[9] McDonagh EM, Wassenaar C, David SP, Tyndale RF, Altman RB, Whirl-Carrillo M (2012). PharmGKB summary: very important pharmacogene information for cytochrome P-450, family 2, subfamily A, polypeptide 6. Pharmacogenet Genomics..

[10] Buchwald J, Chenoweth MJ, Palviainen T, Zhu G, Benner C, Gordon S (2020). Genome-wide association meta-analysis of nicotine metabolism and cigarette consumption measures in smokers of European descent. Mol Psychiatry..

[11] Loukola A, Buchwald J, Gupta R, Palviainen T, Hällfors J, Tikkanen E (2015). A Genome-Wide Association Study of a Biomarker of Nicotine Metabolism. PLoS Genet..

[12] Baurley JW, Edlund CK, Pardamean CI, Conti DV, Krasnow R, Javitz HS (2016). Genome-Wide Association of the Laboratory-Based Nicotine Metabolite Ratio in Three Ancestries. Nicotine Tob Res..

[13] Patel YM, Park SL, Han Y, Wilkens LR, Bickeböller H, Rosenberger A (2016). Novel Association of Genetic Markers Affecting CYP2A6 Activity and Lung Cancer Risk. Cancer Res..

[14] Baurley JW, McMahan CS, Ervin CM, Pardamean B, Bergen AW (2018). Biosignature Discovery for Substance Use Disorders Using Statistical Learning. Trends Mol Med..

[15] El-Boraie A, Taghavi T, Chenoweth MJ, Fukunaga K, Mushiroda T, Kubo M (2020). Evaluation of a weighted genetic risk score for the prediction of biomarkers of CYP2A6 activity. Addict Biol..

[16] El-Boraie A, Chenoweth MJ, Pouget JG, Benowitz NL, Fukunaga K, Mushiroda T (2021). Transferability of ancestry-specific and cross-ancestry CYP2A6 activity genetic risk scores in African and European populations. Clin Pharmacol Ther..

[17] Kolonel LN, Henderson BE, Hankin JH, Nomura AM, Wilkens LR, Pike MC (2000). A multiethnic cohort in Hawaii and Los Angeles: baseline characteristics. Am J Epidemiol..

[18] Stram DO, Hankin JH, Wilkens LR, Pike MC, Monroe KR, Park S (2000). Calibration of the dietary questionnaire for a multiethnic cohort in Hawaii and Los Angeles. Am J Epidemiol..

[19] Patel YM, Stram DO, Wilkens LR, Park SSL, Henderson BE, Le Marchand L (2015). The contribution of common genetic variation to nicotine and cotinine glucuronidation in multiple ethnic/racial populations. Cancer Epidemiol Biomarkers Prev..

[20] Murphy SE, Park SSL, Thompson EF, Wilkens LR, Patel Y, Stram DO (2014). Nicotine N-glucuronidation relative to N-oxidation and C-oxidation and UGT2B10 genotype in five ethnic/racial groups. Carcinogenesis..

[21] Donny EC, Denlinger RL, Tidey JW, Koopmeiners JS, Benowitz NL, Vandrey RG (2015). Randomized Trial of Reduced-Nicotine Standards for Cigarettes. N Engl J Med..

[22] Derby KS, Cuthrell K, Caberto C, Carmella SG, Franke AA, Hecht SS (2008). Nicotine metabolism in three ethnic/racial groups with different risks of lung cancer. Cancer Epidemiol Biomarkers Prev..

[23] Swan GE, Benowitz NL, Jacob P, Lessov CN, Tyndale RF, Wilhelmsen K (2004). Pharmacogenetics of nicotine metabolism in twins: methods and procedures. Twin Res..

[24] Dempsey D, Tutka P, Jacob P, Allen F, Schoedel K, Tyndale RF (2004). Nicotine metabolite ratio as an index of cytochrome P450 2A6 metabolic activity. Clin Pharmacol Ther..

[25] Swan GE, Hudmon KS, Jack LM, Hemberger K, Carmelli D, Khroyan TV (2003). Environmental and genetic determinants of tobacco use: methodology for a multidisciplinary, longitudinal family-based investigation. Cancer Epidemiol Biomarkers Prev..

[26] Baurley JW, Edlund CK, Pardamean CI, Conti DV, Bergen AW (2016). Smokescreen: a targeted genotyping array for addiction research. BMC Genomics..

[27] Browning BL, Tian X, Zhou Y, Browning SR (2021). Fast two-stage phasing of large-scale sequence data. Am J Hum Genet..

[28] Raj A, Stephens M, Pritchard JK (2014). fastSTRUCTURE: variational inference of population structure in large SNP data sets. Genetics..

[29] Josse J, Husson F (2016). missMDA: A Package for Handling Missing Values in Multivariate Data Analysis. J Stat Softw..

[30] Vinzi VE, Chin WW, Henseler J, Wang H, et al. Handbook of partial least squares. vol. 201. Berlin: Springer; 2010.

[31] Huber PJ. Projection pursuit. Ann Stat. 1985;13(2):435–75.

[32] Zou H, Hastie T (2005). Regularization and variable selection via the elastic net. J R Stat Soc Ser B (Stat Methodol)..

[33] Awad M, Khanna R. Support vector regression. In: Efficient learning machines. Berlin: Springer; 2015. p. 67-80.

[34] Natekin A, Knoll A (2013). Gradient boosting machines, a tutorial. Front Neurorobotics..

[35] Breiman L (2001). Random forests. Mach Learn..

[36] Rodriguez JD, Perez A, Lozano JA (2009). Sensitivity analysis of k-fold cross validation in prediction error estimation. IEEE Trans Pattern Anal Mach Intell..

[37] Sagi O, Rokach L (2018). Ensemble learning: A survey. Wiley Interdiscip Rev Data Min Knowl Disc..

[38] Buniello A, MacArthur JAL, Cerezo M, Harris LW, Hayhurst J, Malangone C (2019). The NHGRI-EBI GWAS Catalog of published genome-wide association studies, targeted arrays and summary statistics 2019. Nucleic Acids Res..

[39] Chenoweth MJ, Ware JJ, Zhu AZX, Cole CB, Cox LS, Nollen N (2018). Genome-wide association study of a nicotine metabolism biomarker in African American smokers: impact of chromosome 19 genetic influences. Addiction..

[40] Chenoweth MJ, Cox LS, Nollen NL, Ahluwalia JS, Benowitz NL, Lerman C (2021). Analyses of nicotine metabolism biomarker genetics stratified by sex in African and European Americans. Sci Rep..

[41] Sakaue S, Kanai M, Tanigawa Y, Karjalainen J, Kurki M, Koshiba S (2021). A cross-population atlas of genetic associations for 220 human phenotypes. Nat Genet..

[42] Cornelis MC, Kacprowski T, Menni C, Gustafsson S, Pivin E, Adamski J (2016). Genome-wide association study of caffeine metabolites provides new insights to caffeine metabolism and dietary caffeine-consumption behavior. Hum Mol Genet..

[43] Liu M, Jiang Y, Wedow R, Li Y, Brazel DM, Chen F (2019). Association studies of up to 1.2 million individuals yield new insights into the genetic etiology of tobacco and alcohol use. Nat Genet..

[44] Chenoweth MJ, Novalen M, Hawk LW, Schnoll RA, George TP, Cinciripini PM (2014). Known and novel sources of variability in the nicotine metabolite ratio in a large sample of treatment-seeking smokers. Cancer Epidemiol Biomarkers Prev..

[45] Murphy SE (2017). Nicotine Metabolism and Smoking: Ethnic Differences in the Role of P450 2A6. Chem Res Toxicol..

[46] Taghavi T, Novalen M, Lerman C, George TP, Tyndale RF (2018). A Comparison of Direct and Indirect Analytical Approaches to Measuring Total Nicotine Equivalents in Urine. Cancer Epidemiol Biomarkers Prev..

[47] Swan GE, Lessov-Schlaggar CN, Bergen AW, He Y, Tyndale RF, Benowitz NL (2009). Genetic and environmental influences on the ratio of 3’hydroxycotinine to cotinine in plasma and urine. Pharmacogenet Genomics..

[48] Perez-Paramo YX, Watson CJW, Chen G, Lazarus P (2022). CYP2C19 plays a major role in the hepatic N-oxidation of cotinine. Drug Metab Dispos..

[49] Perez-Paramo YX, Chen G, Ashmore JH, Watson CJW, Nasrin S, Adams-Haduch J (2019). Nicotine-N’-Oxidation by Flavin Monooxygenase Enzymes. Cancer Epidemiol Biomarkers Prev..

[50] Koopmans AB, Braakman MH, Vinkers DJ, Hoek HW, van Harten PN (2021). Meta-analysis of probability estimates of worldwide variation of CYP2D6 and CYP2C19. Transl Psychiatry..

[51] Sosnoff CS, Caron K, Akins JR, Dortch K, Hunter RE, Pine BN (2021). Serum Concentrations of Cotinine and Trans-3’-Hydroxycotinine in US Adults: Results From Wave 1 (2013–2014) of the Population Assessment of Tobacco and Health Study. Nicotine Tob Res..

