# Supplementary material for: Predicting nicotine metabolism across ancestries using genotypes
Source: BMC Genomics. 2022 Sep 21;23:663. doi: 10.1186/s12864-022-08884-z (PMC9490935; doi:10.1186/s12864-022-08884-z)
Supplement: Supplementary file 1 — Additional file 1. Supplementary Figures. [file 12864_2022_8884_MOESM1_ESM.pdf]

## Supplementary Figures

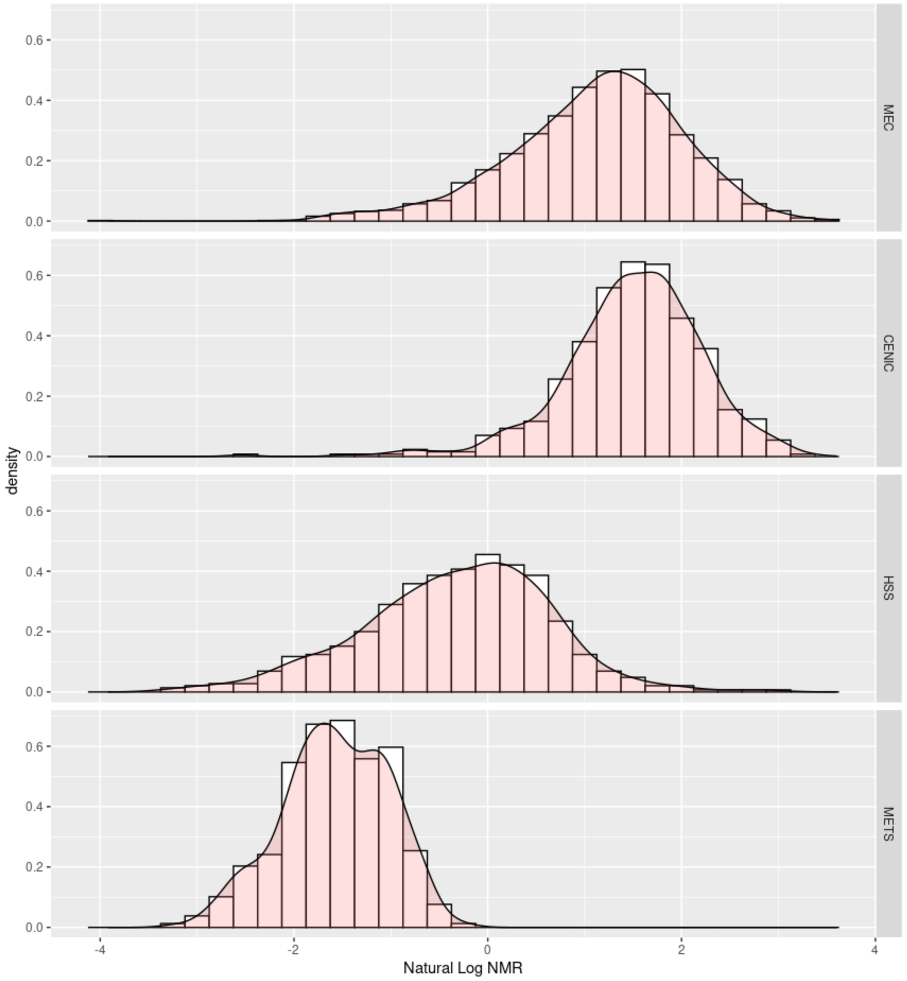


**Figure S1** Distribution of the natural log nicotine metabolite ratio by study. The MEC and CENIC NMR measures were total *trans*-3'-hydroxycotinine (3HC) to free cotinine (COT) in urine. The HSS NMR measure was total 3HC to total COT in urine. The METS NMR measure was 3HC to free COT in plasma or saliva six hours after administration of a fixed dose of deuterium-labeled nicotine and cotinine.

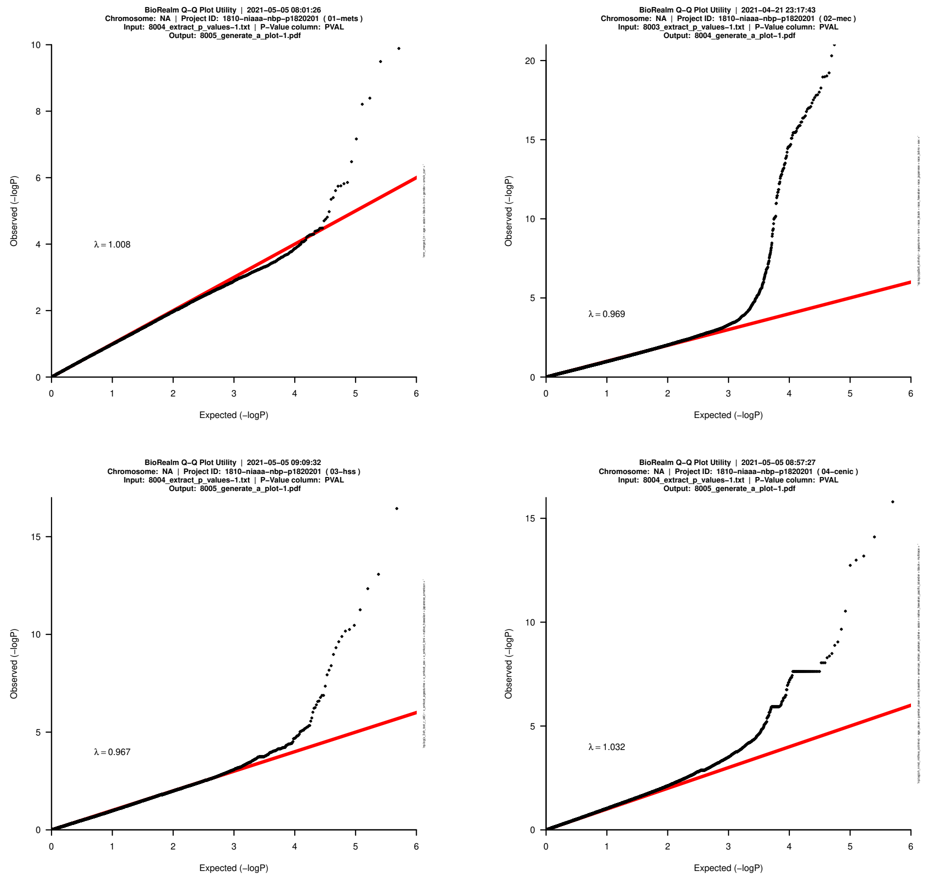


**Figure S2** Observed versus expected distribution of marginal p-values from four genome wide association scans of NMR. Studies from left to right: METS, MEC, HSS, and CENIC. Each model is adjusted for age, sex, BMI, race, and 50 principal components. There is no evidence of systematic inflation or deflation of the p-values. The tails of the distributions represent genetic variants nominated as candidate variables for NMR prediction models.

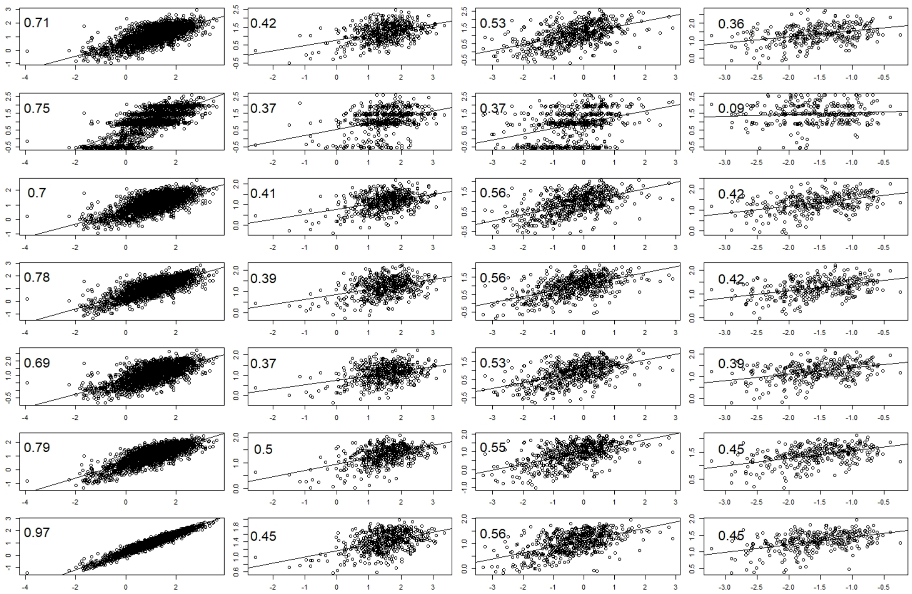


**Figure S3** Plots of predicted versus observed NMR values by four studies (columns) and seven machine learning models (rows). The first column present scatterplots for the training dataset (MEC). The next three columns are the results for the validation datasets (CENIC, HSS, and METS). The models from top to bottom are partial least squares (PLS), project pursuit (PPR), elastic net (ENet), support vector machine with a linear kernel (SVM-L), support vector machine with a radial basis function kernel (SVM-R), gradient boosting machine (GBM), and random forests (RF). The seven models have similar performances in the training data, and thus have equal weights in the ensemble model. It is important to note that the distributions of NMR differ across the studies due to differences in how NMR was collected, measured, and patient characteristics. Yet, correlations are still strong in applying the models trained in MEC to the other datasets.

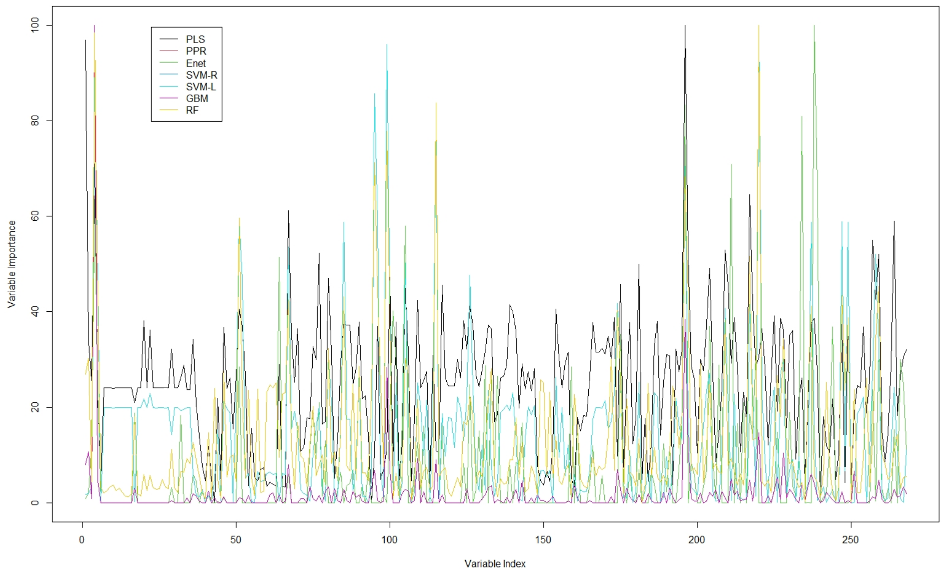


**Figure S4** Candidate variable importance for seven machine learning models trained on the MEC dataset. The 263 genomic variables and covariates considered by each modeling approach are indexed along the x-axis. The influence each variable has on NMR is provided on a scale of 0 to 100 for each model, with higher variable importance indicating greater influence on predicted NMR. There was concordance among the models on the importance of many variables, yet diversity among the models. This property is desirable for ensemble predictions as each component model is using different features of the data to yield similar overall prediction performances.
