# Supplementary material for: Predicting nicotine metabolism across ancestries using genotypes
Source: BMC Genomics. 2022 Sep 21;23:663. doi: 10.1186/s12864-022-08884-z (PMC9490935; doi:10.1186/s12864-022-08884-z)
Supplement: Supplementary file 2 — Additional file 2. Supplementary Table. [file 12864_2022_8884_MOESM2_ESM.pdf]

Supplementary Tables

Table S1 Mean genomic ancestry proportions by self-reported race. Genomic ancestries were estimated by applying `fastSTRUCTURE` to genotypes for 5516 ancestry informative markers extracted from each study and 1000 Genomes Project samples with known population labels. Ancestry proportions were averaged for each self-reported race.

	Estimated African Ancestry	Estimated Asian Ancestry	Estimated European Ancestry
African American (N=520)	0.78	0.01	0.21
American Indian/Alaskan Native (N=5)	0.00	0.21	0.79
Asian American (N=57)	0.00	0.98	0.02
Japanese American (N=865)	0.00	0.98	0.02
Latino (N=453)	0.04	0.38	0.57
Multirace (N=25)	0.24	0.08	0.69
Native Hawaiian/Pacific Islander (N=503)	0.02	0.63	0.35
White (N=1221)	0.01	0.02	0.97
